# Diagnostic Evasion of Highly-Resistant Microorganisms: A Critical Factor in Nosocomial Outbreaks

**DOI:** 10.3389/fmicb.2017.02128

**Published:** 2017-11-03

**Authors:** Xuewei Zhou, Alexander W. Friedrich, Erik Bathoorn

**Affiliations:** Department of Medical Microbiology, University Medical Center Groningen, University of Groningen, Groningen, Netherlands

**Keywords:** diagnostic evasion, outbreaks, HRMO, signaling networks, antibiotic resistance

## Abstract

Highly resistant microorganisms (HRMOs) may evade screening strategies used in routine diagnostics. Bacteria that have evolved to evade diagnostic tests may have a selective advantage in the nosocomial environment. Evasion of resistance detection can result from the following mechanisms: low-level expression of resistance genes not resulting in detectable resistance, slow growing variants, mimicry of wild-type-resistance, and resistance mechanisms that are only detected if induced by antibiotic pressure. We reviewed reports on hospital outbreaks in the Netherlands over the past 5 years. Remarkably, many outbreaks including major nation-wide outbreaks were caused by microorganisms able to evade resistance detection by diagnostic screening tests. We describe various examples of diagnostic evasion by several HRMOs and discuss this in a broad and international perspective. The epidemiology of hospital-associated bacteria may strongly be affected by diagnostic screening strategies. This may result in an increasing reservoir of resistance genes in hospital populations that is unnoticed. The resistance elements may horizontally transfer to hosts with systems for high-level expression, resulting in a clinically significant resistance problem. We advise to communicate the identification of HRMOs that evade diagnostics within national and regional networks. Such signaling networks may prevent inter-hospital outbreaks, and allow collaborative development of adapted diagnostic tests.

## Introduction

Diagnostic screening provides hospitals a level of immunity to antibiotic resistance. When highly resistant microorganisms (HRMOs) are detected, transmission can be limited by treating the patient with isolation precautions. In addition, the carriage of HRMOs can be suppressed by antibiotic treatment or, in case of methicillin resistant *Staphylococcus aureus* (MRSA), even be eradicated. If the introduction of HRMOs in hospitals remains undetected, these bacteria can disseminate from patient-to-patient, and the mobile genetic elements carrying resistance genes can horizontally transfer from species-to-species. Thus, the epidemiology of nosocomial resistance may strongly be affected by our diagnostic screening strategies. Moreover, we postulate that evasion of diagnostic resistance screening could be considered as a critical factor for infection of hospitals with antibiotic resistance elements, similar to the concept that immune evasion is a critical factor of pathogens to infect the human host.

The Netherlands is a high-resource country. Surveillance on HRMOs is extensive in Dutch hospitals. For this, the Dutch situation is very proficient to observe effects of diagnostic screening on the characteristics of HRMOs that cause nosocomial outbreaks. The Dutch Society for Medical Microbiology (NVMM) provides guidelines for the detection of the HRMOs (Working group members NVMM, 2012). For the detection of HRMOs such as carbapenemase-producing Enterobacteriaceae (CPE), vancomycin-resistant enterococci (VRE), extended spectrum beta-lactamase (ESBL) – producing bacteria and MRSA, selective broth and/or selective media are used. Nosocomial outbreaks with HRMO are reported to “Hospital Acquired Infection and Antimicrobial Resistance Monitoring Group”, and the reports are communicated to clinical microbiologists. We searched these reports for outbreaks with micro-organisms harboring resistance mechanisms that were able to evade detection by routine diagnostics. In addition, we evaluated data from our hospital, and searched literature for outbreaks to assess the importance of diagnostic evasion. We here present the most explicit examples of CPE, VRE, ESBL-producing bacteria and MRSA outbreaks caused by isolates harboring diagnostic-evasive resistance mechanisms.

### Diagnostic Evasion by CPE

In the Netherlands, the national laboratory guideline recommends the following screening strategy for the detection of HRMOs: a screening step, a genotypic confirmation step and an optional phenotypic confirmation step (Cohen Stuart et al., 2010; Working group members NVMM, 2012). According to this guideline, *Enterobacteriaceae* with an MIC for meropenem ≥ 0.50 mg/L, or imipenem ≥ 2.0 mg/L should be evaluated by molecular tests for carbapenemase gene detection. Optional phenotypic tests, which include the modified Hodge test, and tests based on inhibition of metallo-betalactamases by EDTA, and Class A carbapenemases by phenyl-boronic acid, may be used if genotypic confirmatory tests are not immediately available. Newer tests for non-genotypic detection of CPE include the carba-NP test, carbapenem-inactivation method (CIM-test), and immunochromotographic tests (Nordmann et al., 2012; van der Zwaluw et al., 2015; Dortet et al., 2016; Literacka et al., 2017). Genotypic confirmation comprises PCR and sequence based methods. Next-gen-sequencing facilities are increasingly accessible for routine diagnostic laboratories. This allows whole-genome sequence-based carbapenemase gene detection. In addition, specific primer/probe combinations for unique markers of an outbreak strains may be designed for high-throughput diagnostics to control outbreaks (Deurenberg et al., 2017).

Despite this huge arsenal of CPE-detection methods, CPEs are still able to evade our diagnostic screening strategies. In the Netherlands, an inter-hospital outbreak with OXA-48-producing *Enterobacteriaceae* from 2009 to 2011 has been reported (Dautzenberg et al., 2014). The outbreak had been uncontrolled for 2 years. The plasmids carrying *bla*_OXA-48_ had disseminated to 15 (sub)- species. Predominantly OXA-48-producing *Escherichia coli* and *Klebsiella pneumoniae* isolates were detected. Heterogeneity in resistance to carbapenems within, and across the OXA-48-producing species was observed. All OXA-48-producing *E. coli* isolates had meropenem MICs of <1 mg/L, a concentration that is commonly used in screening plates, whereas the meropenem susceptibility breakpoint for meropenem is 2 mg/L according to EUCAST (EUCAST, 2017). In addition, if the OXA-48 was not co-expressed with an ESBL gene, no hydrolysis of 3th generation cephalosporins was detected in the majority of isolates. These diagnostic stealth-features have undoubtedly contributed to the magnitude of this outbreak.

The emergence and spread of OXA-48 producing CPEs have been reported in several countries in Europe (Grundmann et al., 2017). The outbreaks concerned predominantly *K. pneumoniae* clones. A successful *K. pneumoniae* clone carrying OXA-48 is ST11, reported in many countries (Lee et al., 2016), amongst others in Greece (Voulgari et al., 2013), Spain (Oteo et al., 2015), and Belgium (De Laveleye et al., 2017). Other clones associated with OXA-48 are ST14, ST15, ST101, SST147, and ST405 (Liapis et al., 2014; Oteo et al., 2015; Lee et al., 2016). In a Belgian multi-center study, less than 50% of CPEs were carbapenem non-susceptible (De Laveleye et al., 2017).

Given the fact that OXA-48 is difficulty to detect, there is a need to adapt surveillance strategies to detect CPEs. The EUCAST-guideline advises to screen for CPE if isolates have a MIC to meropenem > 0.12 mg/L (EUCAST, 2017). Unfortunately, widely used automated susceptibility testing (AST) systems do not detect MICs below 0.5 mg/L. The meropenem MIC distribution of OXA-48-producing *Enterobacteriaceae*, however, shows a peak at MIC = 0.25 mg/L (Fattouh et al., 2015). These isolates will remain undetected if screened by AST only.

When using screening cut-off MICs for CPE detection, which are lower than the susceptibility cut-offs, the sensitivity is still just 80% (Huang et al., 2014). Mainly OXA-48-, and some VIM-producers would remain undetected using meropenem screening cut-offs. Since carbapenem-resistant isolates are usually send to reference centers for CPE detection, this may result in an underestimation of true prevalence numbers (Kaase et al., 2016). In our hospital, we use both culture on screening agars and carbapenemase gene detection directly on rectum samples in patients with a high risk on CPE-carriage to increase the sensitivity of surveillance cultures (Bathoorn et al., 2013). Direct screening of rectal swabs for carbapenemases by real-time PCR performed on enrichment broth showed a higher sensitivity than culturing on selective agar plates (Singh et al., 2012). However, relying on genotypic tests alone may also be a pitfall. For instance, molecular panels for detection of CPE may have a limited number of carbapenemase gene targets. CPEs that are not detected by the panel may have an evolutionary advantage caused by the limitations of this diagnostic method.

### Diagnostic Evasion by VRE

A second example of successful diagnostic evasion by HRMOs is the nationwide emergence of nosocomial outbreaks with vancomycin-resistant *Enterococci* (VRE) in the Netherlands. In the period 2012–2014, 26 outbreaks with VRE have been reported, including reports of local and inter-hospital transmissions (van der Bij et al., 2015). Outbreaks predominantly occurred with VanA- and VanB-type *Enterococcus faecium*, that confer resistance to glycopeptides. *VanB* VRE can easily remain undetected by culturing in routine diagnostics. In addition to the fact that fecal VRE carriage often is detected in very low amounts, vancomycin resistance in *vanB* VRE is not always expressed. These diagnostic challenges have been an important factor in the ongoing transmission of VRE in hospitals in the Netherlands. Several phenotypic screening methods, such as the use of chromogenic agars, have been suggested to identify *vanB* VRE with varying vancomycin MICs (Klare et al., 2012). However, VRE suspected colonies growing on Chrome-agars may test vancomycin susceptible in routine AST systems despite positive genotypic confirmation of *vanB*. This could lead to an unnoticed and uncontrolled spread of *vanB* VRE.

In our hospital, patients are screened on a PCR-based method for VRE on admission at the intensive care unit and if patients are transferred from or recently have been admitted in another hospital in the Netherlands or a foreign hospital. If an unexpected VRE case is found, screening is performed in those patients who are at risk of VRE transmission.

**FIGURE 1:**
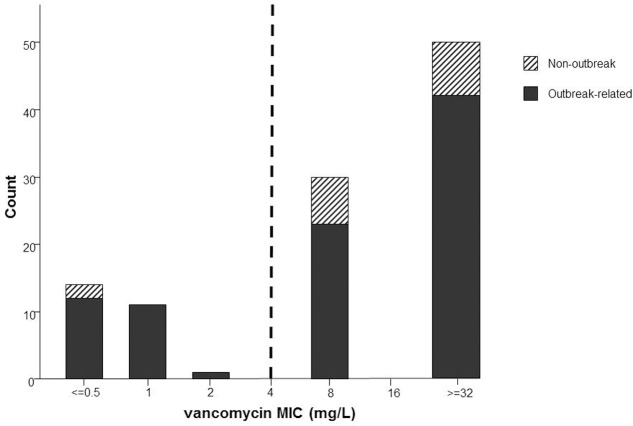
Number of first VRE (all *vanB Enterococcus faecium*) isolates from patients during 2013–2016 and their corresponding MIC values. The dashed line represents the vancomycin susceptibly breakpoint of 4 mg/L.

We have reviewed VRE data from 2013 to 2016 in our own hospitals. We searched for all VRE positive patients and selected their first VRE sample. A total of 106 patients were found, all isolates were *vanB E. faecium.* Of these *vanB* VREs, 26 isolates (24.5%) were tested vancomycin-susceptible by Vitek2 (bioMérieux) according to the EUCAST susceptibility breakpoint of ≤4 mg/L (European Committee on Antimicrobial Susceptibility Testing, 2014). Vancomycin 5 μg paperdisks (Becton Dickenson) were used to phenotypically detect the resistance mechanism, which showed an hazy edge also in the *vanB* positive vancomycin-susceptible isolates. Of these 26 isolates, 24 were outbreak related (92.3%). The two non-outbreak related isolates in the vancomycin-susceptible group were found in a patient transferred from another Dutch hospital and in a patient transferred from a foreign hospital. The other 80 isolates (75.5%) were tested resistant to vancomycin. Of these, 65 isolates (81.3%) were outbreak related (**Figure 1**). The 15 non-outbreak related isolates in this group were from the surveillance cultures of patients transferred from hospitals abroad (*n* = 1), patients transferred from other Dutch hospitals (*n* = 2), in patients admitted to the ICU (*n* = 8), and in clinical samples (*n* = 4). Noticeably, among these 80 patients with vancomycin-resistant *vanB*, we also detected *vanB* positive vancomycin-susceptible *E. faecium* isolates in follow-up samples from 13 patients. These results are in line with reports in literature. A VRE outbreak in a neonatal ICU in Germany has been reported, in which even 55% of the *vanB* positive VRE isolates were tested vancomycin susceptible (Werner et al., 2012). These data show the possible pitfalls in detecting *vanB* VRE in a significant population when only using phenotypic screening tests.

Pitfalls in detecting *vanA* VRE have been described due to an altered phenotype of *vanA* VRE. The expression of teicoplanin resistance can be heterogenous conferring into a VanB phenotype (Park et al., 2008). Moreover, isolates can even test vancomycin susceptible due to a silenced *vanA* gene which can easily lead to uncontrolled outbreaks (Gagnon et al., 2011; Sivertsen et al., 2016).

In a multicenter study, the EUCAST disk diffusion method performed significantly better than the Vitek2 system for the detection of clinical enterococci isolates with low and medium level vancomycin resistance (Hegstad et al., 2014). For rapid detection of VRE carriage, diagnostic strategies using selective enrichment broths and molecular detection can be used to increase the sensitivity of diagnostic procedures (Zhou et al., 2014). Based on above findings, genotypic testing of invasive vancomycin-susceptible enterococci by PCR can be advised. All three diagnostic strategies are being used in our routine diagnostic laboratory.

### Diagnostic Evasion by MRSA

To detect MRSA carriage, the Dutch laboratory guideline recommends to take samples of the throat, nose, and perineum (Working group members NVMM, 2012). Additional body sites should be sampled depending on clinical signs such as wounds, productive cough, skin lesions, or indwelling catheters. To optimize the sensitivity of the cultures, incubation in relatively non-selective enrichment salt-only broths is recommended, followed by culturing for 48 h on selective MRSA screening agars. Additional rapid molecular test are recommended in case of urgency.

In the Netherlands, patients with risk factors for MRSA–carriage such as recent hospitalization abroad, or farm workers at pig farms, cattle farms, or poultry farms are treated upon admission in strict isolation until rapid PCR-based diagnostics are negative. In case of MRSA carriage, patients are treated in isolation and MRSA eradication therapy can be started. This is known as the search and destroy policy (Wertheim et al., 2004). However, PCR-based diagnostics for screening alone would not detect all cases of MRSA-carriage. In a meta-analysis, a sensitivity of 92.5% for the overall pooled PCR estimate has been reported, with a high level of heterogeneity among the studies (Luteijn et al., 2011). PCR-based false negative MRSA results are in our experience usually in patients with a low-level carriage of MRSA. In these cases, culture on chromogenic agar after incubation in broth is more sensitive. In our hospital we use the GeneXpert, an automated PCR-based method to detect MRSA. The lower detection limit for the Xpert MRSA SA nasal assay is about 70 colony forming units (CFUs)/sample according to the manufacturer.

A second reason for failure to detect MRSA is that sporadic Staphylococcal Cassette Chromosome mec (SCCmec)-cassette subtypes, which are a common target in commercial tests, may not be detected by PCR. There is a high diversity in SCCmec-cassettes: already 11 SCCmec-types and numerous subtypes have been designated (Liu et al., 2016). The detection of SCCmec by PCR-based tests is still improving, and the coverage has expanded over the recent years. However, since there may be shifts in common lineages, we should be aware of sporadic nosocomial MRSA that may emerge as successful clones, and are undetectable by commercial tests (Kinnevey et al., 2014). Variety in the *mecA/mecC* target may also result in failure of MRSA detection by PCR. For instance, MRSAs with the divergent homolog *mecA* (*mecA*_LGA251_) would not be detected by the Xpert MRSA assays (Garcia-Alvarez et al., 2011).

False-negative PCR results may have a considerable impact in hospitals. Since patients are discharged from strict isolation after negative PCR results, the isolate has an opportunity to spread until the MRSA is identified by culture and the patient is in strict isolation again. To prevent further spread, contact investigations among patients in the same room and health care workers are performed in these cases in the Netherlands. Since PCR-based detection is not reliable in screening for such isolates, the investigation of contacts has to be performed by culture, which delays the time to detection of secondary transmissions.

Not only PCR-based diagnostics, but also culture-based detection may be evaded by MRSA. In 2014, clinical microbiologists were alerted by a report of the monitoring group on an outbreak with a MRSA strain that could easily be missed by routine diagnostics. Although the numbers of transmissions were largely reduced, total control of the outbreak was difficult due to detection problems using conventional culturing. The *mecA*-positive isolate was difficult to culture as the oxacillin MIC was low, ranging from 0.5 to 6.0 μg/mL. Growth on ChromID^TM^ MRSA agar (bioMérieux) plates was strongly inhibited. We tested the outbreak isolate in our own laboratory and found a more then 10-fold decrease in colony numbers if cultured on ChromID^TM^ MRSA plates compared to blood agar, resulting in a detection limit on ChromID^TM^ MRSA below 0.5 × 10^3^ colonies/100 μL. Molecular testing and prolonged subculturing in broths was advised to detect this isolate.

### Diagnostic Evasion by ESBL

Extended spectrum beta-lactamase-detection can be complicated in natural AmpC-producers such as *Citrobacter freundii, Enterobacter* spp., *Hafnia alvei, Morganella morganii, Serratia* spp. and *Providencia* spp, since it mimicks their natural resistance pattern. Antibiotics can select for mutants with derepressed AmpC expression, resulting in resistance to cephalosporins during therapy. Thus, antibiotic treatment with cephalosporins is not recommended (Livermore et al., 2004). Presence of natural AmpC alone is no condition for HRMO and infection prevention measures.

However, in 2015, several outbreaks in various hospitals in the Netherlands were reported with natural AmpC-producing *Enterobacteriaceae* that acquired additional ESBL genes. This has no consequences for antibiotic therapy choices, however, infection prevention measures need to be taken.

This combined “AmpC-plus-ESBL” phenotype is difficult to distinguish from derepressed-AmpC wild-type resistance. The Dutch laboratory guideline recommend to use cefotaxim and/or ceftazidim to screen for ESBLs with cut-off MIC values for both cephalosporins of >1 mg/L. This screening strategy is also for *Enterobacteriaceae* with natural AmpCs. This leads to many false positive results due to derepressed AmpCs. Phenotypic confirmation based on inhibition ESBL activity by clavulanic acid or cefepime hydrolysis by disk diffusion, Etest or broth microdilution methods is recommended.

Natural AmpC-producing *Enterobacteriaceae* that acquired additional ESBL genes are common in Dutch nosocomial isolates. *Citrobacter freundii* and *Enterobacter cloacae* showed the highest percentages of confirmed ESBL co-producers: 3% of *Citrobacter freundii* (total *n* = 9.432), and 2% of *Enterobacter cloacae* (*n* = 28.027) were recorded by the Dutch national antibiotic resistance surveillance system (ISIS-AR). Microbiologist were explicitly warned for outbreaks with these difficult to detect HRMOs in a report by the monitoring group.

The substantial presence of ESBLs in *Enterobacteriaceae* with natural AmpCs has been underlined in an Asian study (Choi et al., 2007). The ESBLs confer additional resistance to fourth generation cephalosporins, compared to the natural broad-spectrum AmpCs. These isolate may represent a hidden reservoir of ESBL-carrying plasmids, which can transfer across species. Numerous outbreaks with ESBL natural AmpC producers have been reported in international literature (Mezzatesta et al., 2012). Since resistance to 3th generation cephlosporins is very common in natural AmpC producers that do not carry ESBLs (Jacobson et al., 1995), the dissemination of ESBL-carrying isolates in hospitals may remain unnoticed.

## Implications and Future Directions

We observed that highly-resistant microorganisms adapt to evade screening strategies. One can consider this process as a prey that evolves to escape from predators. Microbiologists, in their evolutionary role as predators hunting for HRMOs, also have to keep on innovating to update the detection strategies for these micro-organisms that are trying to evade. This may result in an arms race. In evolutionary biology, such an arms race is known as the Red Queens hypothesis (Castrodeza, 1979). The name of the theory is based on a quote from Lewis Carroll’s *Through the Looking-Glass*: “Now, here, you see, it takes all the running you can do, to keep in the same place. If you want to get somewhere else, you must run at least twice as fast as that!”

To run twice at fast, communication within networks of health care professionals is crucial. In our perspective, we described examples of how Dutch clinical microbiologist were alarmed by a national monitoring group on successful HRMOs that evade routine screening tests. Specific recommendations to adjust diagnostic strategies to detect these pathogens were provided. Additionally, rapid communication within regional networks is of utmost importance. Inter-hospital patient traffic is highest between hospitals in the same regions. As a consequence, hospitals within the same region are at immediate risk of introduction of HRMOs that evade diagnostics and cause outbreaks. We recommend to identify your region of hospitals that are most connected by patient traffic, and set-up communication networks to alarm for difficult to detect HRMO’s. Experiences and adjusted diagnostic screening tests should be shared within these networks. Such a regional approach has successfully been applied in the control of MRSA in the Dutch-German cross-border region (Jurke et al., 2013).

We should be aware of the impact of our diagnostics on the introduction and dissemination of resistance elements in our hospitals. The Government of the Netherlands has a national and international mission to combat antimicrobial resistance (AMR). Therefore the NVMM has composed a vision document to maintain the low prevalence of CPE in the Netherlands (Kluytmans et al., 2015). By taking CPE as a biological indicator, it is implicitly assumed that other HRMOs will be included in the combat of AMR. To realize the goals, it is of utmost importance that diagnostic methods are continuously innovated and used.

We are aware that optimizing diagnostic screening will increase costs. On the other hand, our examples have shown that failure of detection by routine diagnostics may lead to uncontrolled outbreaks. These outbreaks can lead to enormous financial expenses; costs may rise up to €1,369 per patient per day (Dik et al., 2016). Moreover, detection of HRMO carriage allows for directed antibiotic treatment of patients developing infections by these HRMOs.

Cost reductions in innovation of diagnostics for screening purposes are foretold to result in nosocomial outbreaks with HRMOs evading our screenings methods. We would be outsmarted by prokaryotes.

## Author Contributions

XZ and EB wrote this perspective article. AF carefully reviewed it.

## Conflict of Interest Statement

The authors declare that the research was conducted in the absence of any commercial or financial relationships that could be construed as a potential conflict of interest.
